# Expression profiling and functional analysis of Toll-like receptors in primary healthy human nasal epithelial cells shows no correlation and a refractory LPS response

**DOI:** 10.1186/s13601-015-0086-3

**Published:** 2015-12-14

**Authors:** J. van Tongeren, K. I. L. Röschmann, S. M. Reinartz, S. Luiten, W. J. Fokkens, E. C. de Jong, C. M. van Drunen

**Affiliations:** Department of Otorhinolaryngology, Academic Medical Center (AMC), Amsterdam, The Netherlands; Department of Cell Biology & Histology, Academic Medical Center (AMC), Amsterdam, The Netherlands

**Keywords:** Nasal epithelial cells, Pattern recognition receptors, Toll-like receptor, Chemokines, Cytokines

## Abstract

**Background:**

Innate immune recognition via Toll-like receptors (TLRs) on barrier cells like epithelial cells has been shown to influence the regulation of local immune responses. Here we determine expression level variations and functionality of TLRs in nasal epithelial cells from healthy donors.

**Methods:**

Expression levels of the different TLRs on primary nasal epithelial cells from healthy donors derived from inferior turbinates was determined by RT-PCR. Functionality of the TLRs was determined by stimulation with the respective ligand and evaluation of released mediators by Luminex ELISA.

**Results:**

Primary nasal epithelial cells express different levels of TLR1-6 and TLR9. We were unable to detect mRNA of TLR7, TLR8 and TLR10. Stimulation with Poly(I:C) resulted in a significant increased secretion of IL-4, IL-6, RANTES, IP-10, MIP-1β, VEGF, FGF, IL-1RA, IL-2R and G-CSF. Stimulation with PGN only resulted in significant increased production of IL-6, VEGF and IL-1RA. Although the expression of TLR4 and co-stimulatory molecules could be confirmed, primary nasal epithelial cells appeared to be unresponsive to stimulation with LPS. Furthermore, we observed huge individual differences in TLR agonist-induced mediator release, which did not correlate with the respective expression of TLRs.

**Conclusion:**

Our data suggest that nasal epithelium seems to have developed a delicate system of discrimination and recognition of microbial patterns. Hypo-responsiveness to LPS could provide a mechanism to dampen the inflammatory response in the nasal mucosa in order to avoid a chronic inflammatory response. Individual, differential expression of TLRs on epithelial cells and functionality in terms of released mediators might be a crucial factor in explaining why some people develop allergies to common inhaled antigens, and others do not.

## Background

The mucosal barrier of the nose forms the first line of defence against air pollutants, airborne allergens, and (non-) pathogenic microorganisms. Epithelial cells are the outer lining of the mucosa of the nasal airway and play, besides their role as passive physical barrier, an important role in orchestrating innate and adaptive immune responses [1–3]. Epithelium can trigger anti-microbial responses by recognizing pathogen-associated molecular patterns (PAMPs) through the sentinel action of pattern recognition receptors (PRRs) like Toll-like receptors (TLRs).

Toll-like receptors are evolutionarily conserved pattern recognition receptors of the innate immune system [4]. Until now, 13 mammalian TLRs have been characterized and for most of the TLRs, except TLR10, TLR12 and TLR13, specific ligands have been identified [5, 6]. The first group of receptors recognize bacterial products. TLR2 is activated by a variety of bacterial lipoproteins, peptidoglycans (PGN), and lipoteichoic acids (LTA), by forming a heterodimer with TLR1 or TLR6 [7, 8]. TLR4 appears to form homodimers and under participation of adaptor molecules like MD-2 and CD14 this TLR recognizes lipopolysaccharide (LPS) from the outer membrane of gram-negative bacteria. Originally thought to be a receptor only for LPS, TLR4 now emerges as a molecule responsible for signalling induced by a broad variety of molecules such as respiratory syncytial virus protein F [9], fungal components [10], or endogenous ligands like heat shock proteins, lung surfactant protein A, and beta-defensin [11–13]. Lastly, TLR5 recognizes bacterial Flagellin. Viral compounds trigger endosome-associated receptors, such as TLR3 by double-stranded (ds) RNA or its synthetic analogon polyinosinic polycytidylic acid (Poly I:C) and viral single stranded (ss) RNA signals via TLR7 and TLR8. DNA-containing CpG motifs are recognized via TLR9 [14, 15].

Although TLRs can be involved in the initiation of adaptive immune responses through their presence on dendritic cells (DCs), they may also indirectly affect the adaptive immune response. Innate immune recognition via TLRs on barrier cells like epithelial cells has been shown to determine the functional properties of tissue-resident DCs, thereby instructing the outcome of antigen-specific immunity [16]. The overall complexity of the contribution of TLRs on immune responses can be influenced by several factors like their relative abundance, their individual expression pattern, or the timing of exposure. For example, stimulation of TLR2 and TLR4 signalling pathways has been shown to both drive [16, 17] and inhibit [18, 19] the development of Th2-mediated allergic inflammation in different experimental mouse models. Moreover, the impact of TLR4 stimulation on allergic inflammation is highly dependent upon the dose of the TLR4 agonist, with high LPS concentrations inducing Th1-responses and low concentrations inducing Th2-polarized inflammatory responses [20].

The LPS-induced pulmonary burden varies between different respiratory compartments and might therefore explain the functional differences between bronchial and alveolar epithelial cells with respect to LPS-dependent cytokine release. It is widely believed that alveolar epithelial cells are unresponsive to LPS due to low or absent expression of TLR4 and/or CD14 or MD-2 [21]. In contrast to this it has been reported that lung epithelial cells do express TLR 1–6, including adaptor molecules like CD14 and MD-2, with bronchial epithelial cells showing CD14-dependent activation of TLR4 and alveolar epithelial cells showing LPS-binding protein (LBP)-dependent inhibition of TLR4 signalling [22, 23], confirming the relevance of co-factors for a proper TLR signalling. Small airway epithelial cells have been shown to express mRNA for the TLRs 1–6 and can respond to various stimuli such as viruses or bacteria resulting in the release of different pro- and anti-inflammatory cytokines and chemokines [24–26].

At present only limited data is available on the expression of TLRs by nasal epithelial cells. Claeys et al. showed constant expression of TLR2 and TLR4 in tissue biopsies from patients with nasal polyposis or chronic rhinosinusitis and in healthy individuals [27]. Isolated nasal epithelial cells from nasal polyps were shown to constitutively express mRNA of all 10 TLRs, with more pronounced expression of TLR 1–6 [28]. Primary nasal polyp epithelial cells express functional TLR3 and TLR4 and release high concentrations of proinflammatory chemokines and cytokines upon stimulation with dsRNA [29]. Until only one study investigated the expression and function of some but not all TLRs on primary nasal epithelial cells from non-allergic, non-diseased individuals specifically [30]. Given the role of TLRs expressed on epithelial cells within the induction of immune responses, the relatively limited knowledge on the expression of TLRs in nasal epithelium, and the protective effect of TLR polymorphism in childhood asthma [31], the aim of this study was to determine the expression and functionality of TLRs expressed in primary nasal epithelial cells from healthy donors.

## Methods

### Patient characteristics

Nasal tissue was obtained from 10 non-allergic ENT patients (defined by negative skin prick test or radioallergosorbent test (RAST)) with septum deviations that required inferior turbinectomy. Patients were between 18 and 65 years of age, were nonsmokers, had not received topical corticosteroids for at least 4 weeks before surgery, and were free of any respiratory tract infections. The study was reviewed and approved by the medical ethical committee of the Academic Medical Center Amsterdam and all patients gave their informed consent.

### Epithelial cell culture

Primary nasal epithelial cells were obtained by digesting nasal turbinates of non-allergic patients with 0.5 mg/ml collagenase 4 (Worthington Biochemical Corp., Lakewood, NJ) for 1 h in Hanks’ balanced salt solution (HBSS; Sigma-Aldrich, Zwijndrecht, The Netherlands). Subsequently, epithelial cells were isolated by magnetic activated cell sorting (MACS), according to the manufacturers instruction (Miltenyi Biotec, Leiden, The Netherlands), resuspended in bronchial epithelial growth medium (BEGM) (Lonza Clonetics, Breda, The Netherlands), and seeded in a 75 ml flask. Culture medium was replaced every other day. Cells were grown to 80 % confluency in fully humidified air containing 5 % CO_2_ at 37 °C.

NCI-H292 human airway epithelial cells (American Type Culture Collection, Mannassas, VA, USA) were cultured in RPMI 1640 medium (Invitrogen, Breda, The Netherland) supplemented with 1.25 mM l-glutamine, 100 U/ml penicillin, 100 μg/ml streptomycin and 10 % (v/v) fetal bovine serum (HyClone, Logan, UT, USA). Cells were grown in fully humidified air with 5 % CO_2_ at 37 °C and sub cultured weekly.

### TLR stimulation experiment

Cells were cultured up to a confluence of 80 % in a six wells plate and incubated for 24 h in IMDM without supplements. Culture medium was removed and cells were stimulated with different TLR-agonists diluted in IMDM or with IMDM alone (control condition) for 24 h. Supernatants were removed after 1, 4, and 24 h and stored for further analysis; cells were used for RNA extraction. Each experiment was performed in triplicate. LPS (*Escherichia coli*), PGN (*Staphylococcus aureus*), and dsRNA (poly(deoxyinosinic-deoxycytidylic acid)) were from Sigma-Aldrich. ssRNA (LyoVec) and flagellin (*Salmonella typhimurium*) were from InvivoGen and the ligands were used at optimal concentrations as determined in a previous dose range finding experiment: PGN: 10 μg/mL, Poly(I:C): 20 μg/mL, LPS 1 μg/mL, Flagellin: 1 μg/mL, and CpG2216: 0.5 μM. As positive controls we used TNF-α (25 ng/mL) and IL-1β (10 ng/mL).

### RNA extraction and Real-time quantitative RT-PCR analysis

PCR was used to validate the differential expression of selected genes. Isolated mRNA (Kit from Macherey–Nagel, Düren, Germany) was transcribed into cDNA using the MBI Fermentas first strand cDNA kit. cDNA transcripts were quantified by real-time quantitative PCR (iCycler iQ MultiColor Real-Time PCR Detection System; Bio-Rad) with specific primers [32] and general SYBR green (Bio-Rad) fluorescence detection. mRNA expression of each sample was normalized to GAPDH. All PCRs have been performed for all participants on 3 biological replicates. Expression changes are presented as 2^−ΔCt^ indicating the difference in threshold cycle between the housekeeping gene GAPDH and the investigated TLR gene.

### FACS analysis

For flow cytometry analysis cells were stained with CD14-PE-Cy7 (1:20, BD Bioscience, Breda, the Netherland), TLR4-APC (1:10, ebioscience, San Diego, USA) or left untreated. Cell numbers were quantified using the BD FACS Cantoll flowcytometer, histograms were generated using flowjo software version 7.6.2 (Treestar Inc, Ashland-OR, USA.

### Determining cytokine and chemokine production by ELISA

Cell free supernatants of stimulated and control treated cells were stored at −20 °C until analysis. Cytokine levels in supernatant of cells were determined by ELISA (IL-6 and IL8, BioSource International Camarillo-CA, USA) or using the xMAP technology (Luminex Corporation, Austin-TX, USA). A Bio-Plex Human Cytokine 17-Plex Panel kit (Bio-Rad, Veenendaal, The Netherlands). Concentrations were calculated from a dilution series of standards using the Luminex software. Lower detection limits are indicated per cytokine.

### Statistical analysis

Assessment of statistical significance for Luminex data was performed using two-tailed Student’s t tests with GraphPad Prism. P values <0.05 were considered significant. Relationships between parameters were assessed using Pearson’s correlation analysis.

## Results

### Baseline expression and functionality of TLR in human nasal epithelial cells

**Fig. 1:**
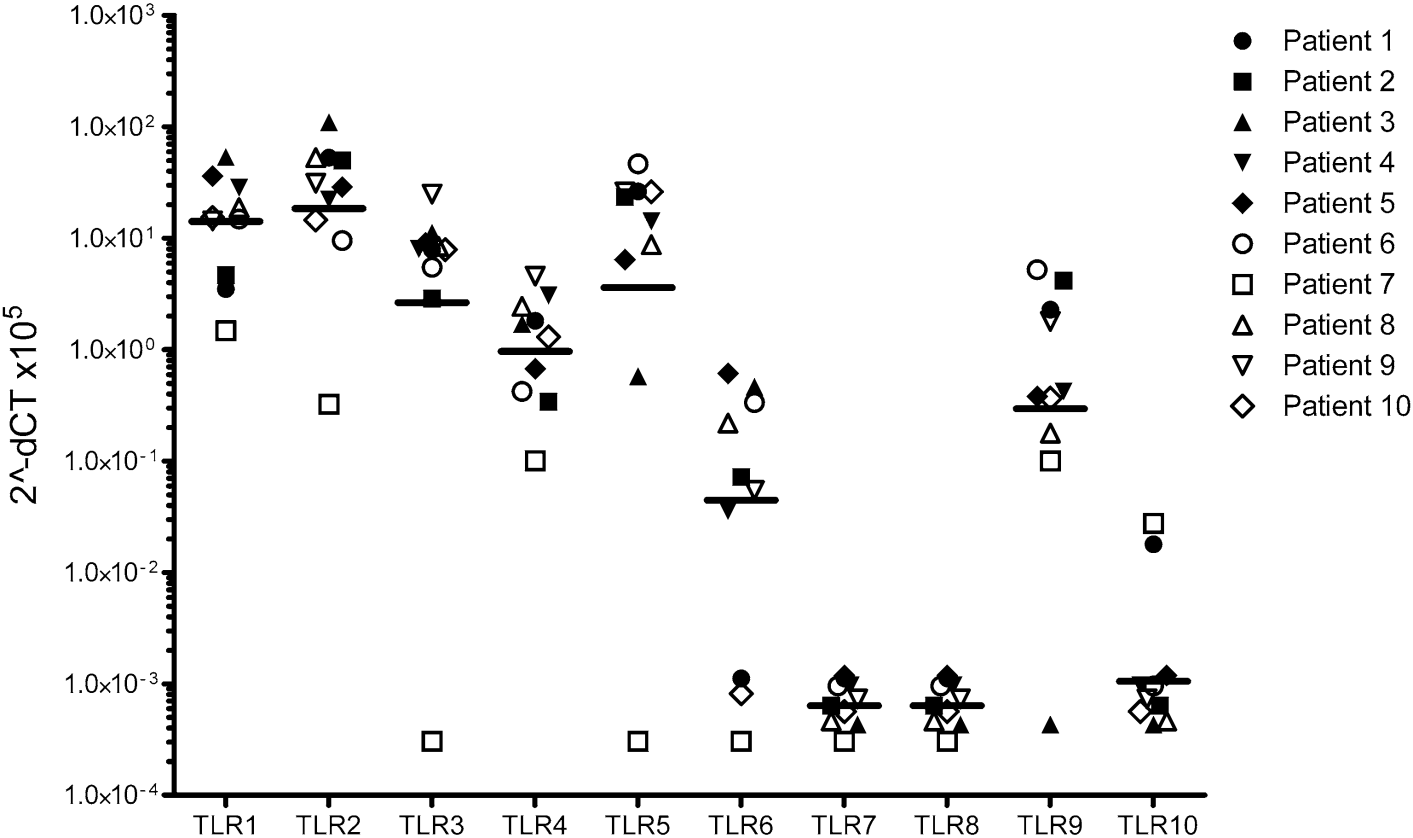
Toll-like Receptor (TLR) mRNA expression by primary nasal epithelial cells from 10 healthy patients undergoing turbinectomy (n = 10). TLR 1-10 mRNA expression was analyzed by quantitative RT-PCR. Results were normalized using GAPDH as endogenous control. Expression changes are presented as 2^−ΔCt^ × 10^5^ indicating the difference in threshold cycle between the housekeeping gene GAPDH and the investigated TLR gene

**Fig. 2:**
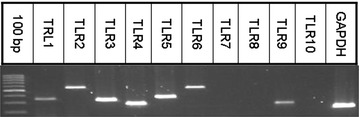
Toll-like Receptor (TLR) mRNA expression by primary nasal epithelial cells from one representative patient. Expression of TLRs was analyzed by quantitative RT-PCR. Products were visualized by agarose gel-electrophoresis in a 2 % agarose gel

As shown in Figs. 1 and 2, primary nasal epithelial cells from healthy donors express TLR1 to TLR6 and TLR9, but not TLR7 and TLR8, and TLR10. Interestingly, we observed a huge individual variability spanning several 10 log-fold differences in the baseline expression of the TLRs to the extent that some healthy individuals do not express TLRs that are expressed by others.

**Fig. 3:**
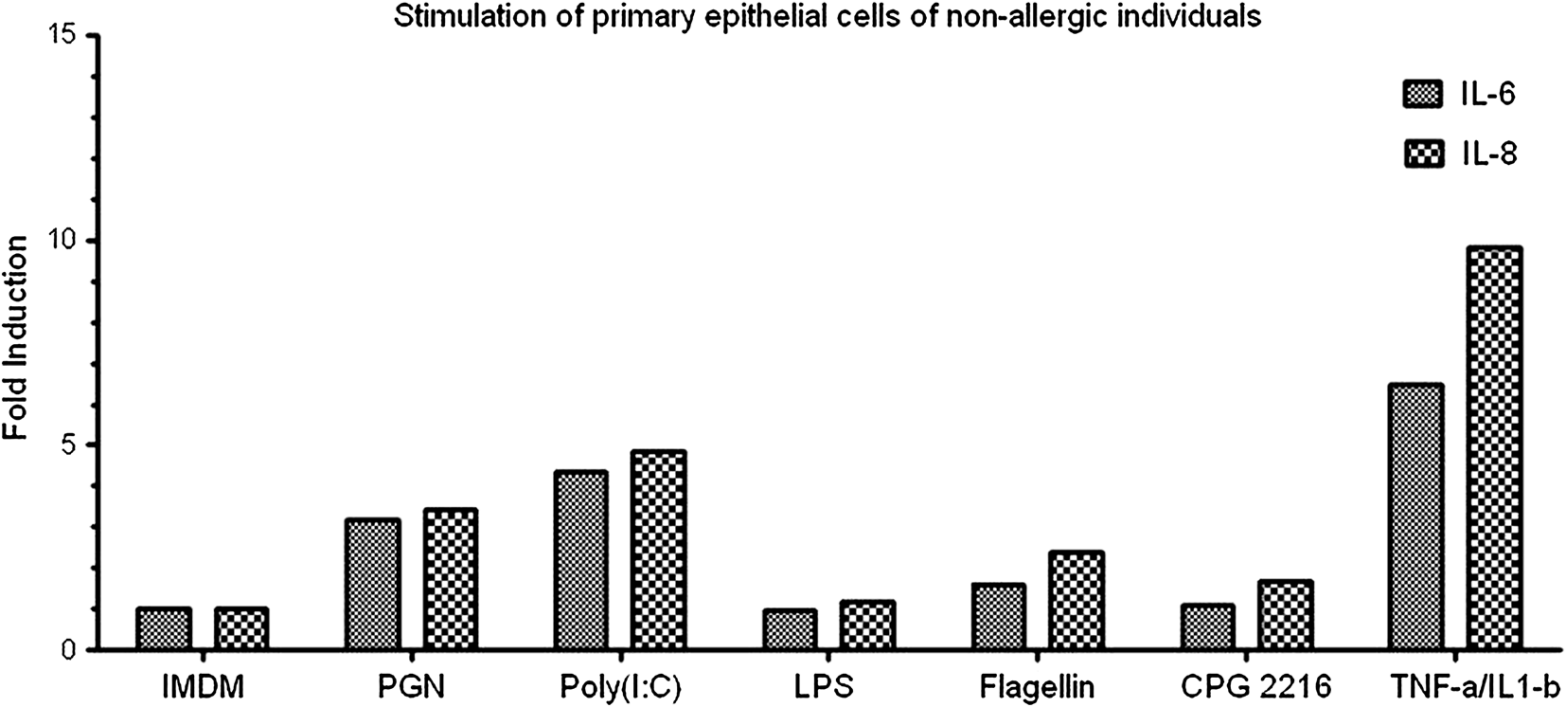
Primary nasal epithelial cells of non-allergic individuals were stimulated for 24 h with different TLR ligands. IL-6 and IL-8 production was measured after 24 h by ELISA. Results from one representative patient are shown as fold induction compared to unstimulated cells

**Fig. 4:**
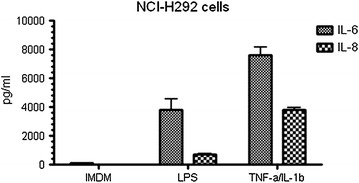
NCI-H292 cells were stimulated for 24 h with LPS (1 μg/ml) and TNF-α (25 ng/ml) and IL-1β (10 ng/ml). Cell free supernatants were analyzed for the release of IL-6 and IL-8 by ELISA

**Fig. 5:**
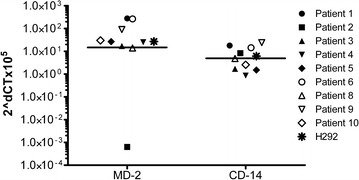
MD-2 and CD14 mRNA expression by primary nasal epithelial cells from healthy patients (n = 9) and NCI-H292 cells. MD-2 and CD14 mRNA expression was analyzed by quantitative RT-PCR. Results were normalized using GAPDH as endogenous control. Expression changes are presented as 2^−ΔCt^ × 10^5^ indicating the difference in threshold cycle between the housekeeping gene GAPDH and the investigated genes

**Fig. 6:**
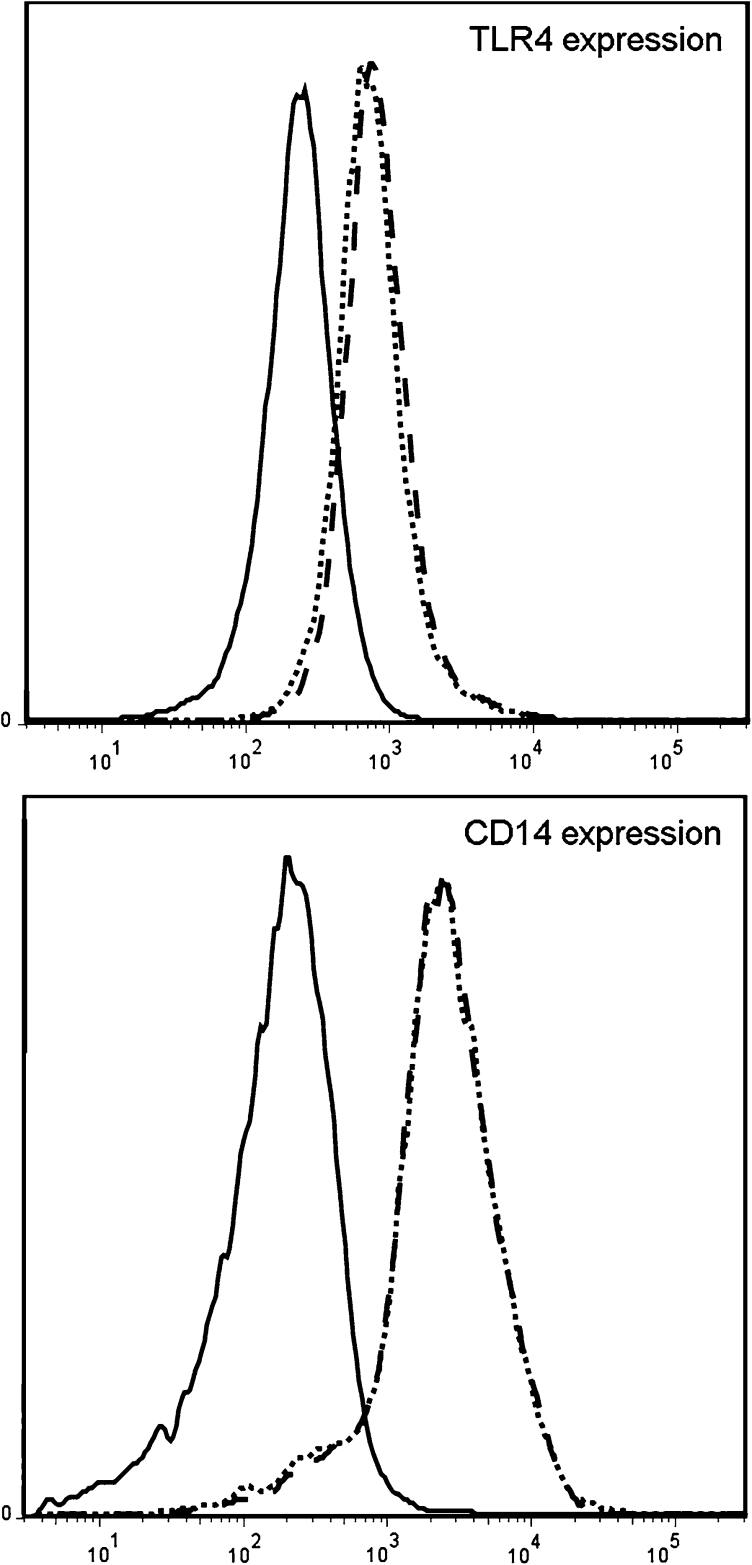
Expression of TLR4 and CD14. Surface expression of TLR4 and CD14 on primary nasal epithelial cells was assessed using flow cytometry. Histograms with solid lines represent controls, spotted lines display surface expression of TLR4 (*upper graph*) or CD14 (*lower graph*) under unstimulated conditions. Histograms with dashed lines illustrate TLR4 (*upper graph*) or CD14 (*lower graph*) expression upon stimulation with LPS (1 μg/mL, 24 h)

In order to determine the functionality of the detected TLRs we stimulated primary nasal epithelial cells with their purified specific TLR ligands and used TNF-α and IL-1β as positive control to show the ability of our epithelial cells to respond to external triggers. As shown in Fig. 3, stimulation of primary heathy epithelial cells by PGN, Poly(I:C), and Flagellin resulted in increased release of IL-6 and IL-8 confirming the biological functionality of TLR2, TLR3, and TLR5 respectively. Remarkably, healthy primary nasal epithelial cells do not seem to respond to TLR4 ligation by LPS (Fig. 3) despite expressing the TLR4 gene and the use of a biological active LPS, as seen by the positive response in the epithelial cell line H292 (Fig. 4). Furthermore, we could show (Figs. 5, 6) surface expression of TLR4 and CD14, and co-expression of the adaptor MD-2 that are indispensable for proper TLR4 signalling [33, 34].

In a next step we analyzed, which additional cytokines and chemokines are released from primary nasal epithelial cells in response to activation by different TLR ligands. As shown in Table 1 the multiplex ELISA showed that stimulation of primary nasal epithelial cells with Poly(I:C) resulted in a significant increased secretion of IL-4, IL-6, RANTES, IP-10, MIP-1β, VEGF, FGF, IL-1RA, IL-2R, and G-CSF. Furthermore, stimulation with PGN resulted in significantly increased production of IL-6, IL-1RA, and VEGF. In contrast to stimulation of epithelial TLR2 and TLR3, and confirming our previous observations, stimulation with a high concentration of LPS did not result in increased secretion of any cytokines or chemokines.

**Table 1:** Primary nasal epithelial cells of 5 non-allergic individuals were stimulated for 24 h with different TLR ligands: TLR2 (PGN: 10 μg/ml), TLR3 (Poly(I:C): 20 μg/ml), TLR4 (LPS 1 μg/ml)

	Cut off value	IMDM		Poly(I:C)		LPS		PGN	
		Mean	SD	Mean	SD	Mean	SD	Mean	SD
IL-1β	20	BD		26	28	BD		BD	
IL1RA	15	198	151	713**	487	219	111	337**	179
IL-2	3	BD		13	15	BD		BD	
IL-4	3	BD		13**	12	BD		BD	
IL-5	2	BD		BD		BD		BD	
IL-6	3	351	331	1793**	1491	264	256	548**	521
IL-7	3	24	36	132	122	34	35	45	44
IL-10	16	BD		BD		BD		BD	
IL-12	8	BD		46	27	BD		BD	
IL-13	4	BD		BD		BD		BD	
IL-15	6	BD		28	27	BD		BD	
IL-17	7	17	19	26	32	11	17	10	20
Eotaxin	6	BD		BD		BD		BD	
FGF basic	15	BD		40**	40	BD		BD	
G-CSF	2	72	95	655**	700	BD		132	179
GMCSF	4	36	74	58	148	18	47	20	48
INF γ	80	BD		BD	16	BD		BD	
IP-10	24	BD		1982**	1666	BD		29	27
MCP 1	6	219	111	414	456	204	107	278	216
MIP1α	2	8	16	1122	2376	4	10	4	11
MIP1β	38	BD		930**	1321	BD		BD	
MIG	4	5	11	23	22	BD		BD	
RANTES	5	19	23	2120**	1516	18	21	25	25
TNFα	10	BD		43	68	BD		BD	
VEGF	9	191	163	482**	508	161	142	262**	189
EGF	1	BD		5	10	BD		1	4
HGF	66	BD		31	28	BD		BD	
IL-2R	7	70	52	632**	491	65	36	99	63
IFNα	2	14	21	39	39	10	18	22	20

Cell free supernatants were analysed using a Luminex array. Concentrations are presented as average of triplicates of 5 different patients in pg/ml. The detection limits are shown as cut off value

*SD* Standard deviation, *BD* below detection level

** P < 0.05

### No correlation between TLR expression levels and level of cytokine release

**Fig. 7:**
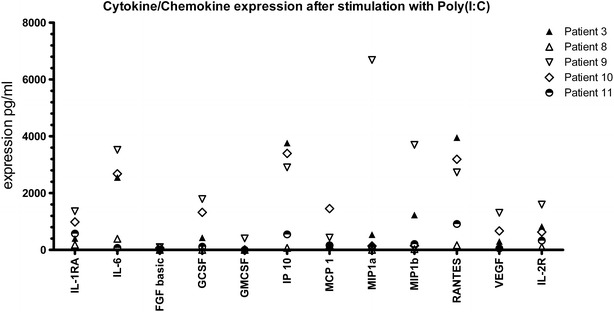
Cytokine and chemokine secretion by stimulated primary nasal epithelial cells. Primary nasal epithelial cells of 5 non-allergic individuals were stimulated for 24 h with the TLR3 agonist Poly(I:C). Cell free supernatants were analysed using a Luminex array. Concentrations are presented as average of triplicates of 5 different patients

Strikingly, our data also revealed large individual differences in cytokine expression patterns. To investigate the functional consequences of this variation we first determined the individual mediator levels of all donors after stimulation with Poly(I:C). As shown in Fig. 7, some individuals seemed to be high responders (individual 3 and 9), while nasal epithelial cells from others (e.g. individual 11) hardly produced any significant levels of the mediators included in the assay used. Furthermore, these induction levels were not related to the expression levels of TLR3 (data not shown).

## Discussion

Epithelial cells are uniquely positioned at the interface between inside and outside of the organism, which makes them perfect candidates for initiating and orchestrating local immune responses. In addition to establishing which TLR receptors are expressed in primary nasal epithelial cells from healthy individuals, our data furthermore suggest that nasal epithelium has developed a delicate response system towards microbial exposures. Firstly, despite the presence of TLR4 and its prime co-stimulatory molecules CD14 and MD-2, nasal epithelium from healthy individuals does not respond to LPS. As the nasal mucosa is constantly exposed to high concentrations of endotoxin, this unresponsiveness could provide a mechanism to dampen the inflammatory response in the nasal mucosa in order to avoid a chronic inflammatory response. Secondly, levels of TLR expression in individuals varies strongly, to the extent that some individuals not express TLRs that others do. Thirdly, not only are the expression levels different between individuals, but independently of these differences, also the response induced by a specific TLR ligand varies strongly between individuals. And finally, the mediator response varies between different TLRs, even when they are thought to act through a common pathway. This complex level of variation in TLR signaling suggests that different healthy individuals may see different environments despite identical exposure. Although it should be noted that our sample size of 10 healthy individuals is relatively small, so that it would be difficult to generalize our conclusions for the general population

The expression of TLRs within the lower respiratory tract has been investigated intensively [22, 24, 25, 35], while data on TLR expression and functionality in nasal epithelial cells from healthy individuals is limited. Our experiments showed that primary nasal epithelial cells from most healthy donors express mRNA for TLR 1–6 and TLR9 and mainly respond to the TLR3 ligand Poly(I:C) and to the TLR2 and TLR5 agonists. The expression of TLR2, 3, and 4 has been shown in nasal epithelial cells derived from nasal polyps, with poly(I:C) inducing the secretion of RANTES, IP-10, IL-8 and GM-CSF [29]. We were able to confirm this outcome and in addition show a consistent and statistically significant up-regulation of IL-2R, VEGF, MIP-1β in all individuals. Closer inspection of our data shows strong up-regulation of other mediators as well (e.g. Mip-1α, MCP1, IL-7). However, as the induced expression of these mediators varies so strongly between individuals this up-regulation does not reach statistical significance. These observations show that healthy individuals differ strongly in their response to external triggers, which will contribute to differences between the ability to fight off viral and bacterial infections. The absence of TLR7 and TLR10 mRNA expression differs from the previous observations of Renkonen [36] and Tengroth [30]. In both previous reports expression levels at the mRNA were low, so that differences in our detection technique (real time PCR) versus microarray [36] or differences in growth conditions [30, 36] may help to explain the observed expression differences for TLR7 and TLR10. Allowing for the specificity of TLR antibodies both TLR7 and TLR10 could be detected by immuno-histochemistry with moderate biological activity for the TLR7 agonist relative to TLR3 activation [30, 36].

The most striking discrepancy between TLR expression and responsiveness we observed for TLR4. Despite the presence of the receptor on the cell surface, the presence of key co-stimulatory molecules (CD14 and MD-2) and a seemingly intact downstream signaling cascade (the cells do response to other TLR stimulations), nasal epithelial cells do not respond to LPS. This unresponsiveness has also been observed in the epithelia of the gut where it was attributed to missing MD-2 expression [37] and in nasal epithelium from polyposis patients by Wang and co-workers [29]. Nasal polyposis epithelium showed a much weaker response to LPS than to polyIC stimulation, indicating that even in diseased tissue the nasal epithelial response to LPS is affected. In contrast, lung or renal epithelia are able to respond to LPS which suggests that the hypo-responsiveness could be an adaptation in epithelia that are exposed to high concentrations of LPS, whereas epithelia that are relative sterile do show a response to LPS.

The functional consequences of responding to TLR ligation are many. Expression of IP-10, MIP-1α, MIP-1β, IL-8 and G-CSF after TLR3 activation contribute to the recruitment and activation of neutrophils or macrophages. Furthermore, IL-8 and RANTES have been shown to be involved in the recruitment and survival of eosinophils [38]. These findings imply a role for TLR3 in the nasal immune response not only in Th1-mediated responses, but also in viral induced allergic exacerbations. This notion would also be in line with our recent observation that many aspects of TLR3 activation of nasal epithelial cells resemble that of activation by house dust mite allergen [39]. The inflammatory features of dsRNA mediated by TLR3 are also thought to contribute to the exacerbation of CRS and nasal polyps during viral infection [40]. TLR4 expression on lung epithelial cells has been shown to be required for DC activation in the lung and for priming of effector T helper response to HDM [16]. In the absence of TLR ligation, lung DCs are minimally active. Binding with the TLR4 ligand LPS leads to enhanced motility and sampling behavior. This response strictly depends on neighboring epithelial cells being triggered by TLR4. In addition, responses to allergens are substantially altered when epithelial cells cannot detect the endotoxin in the allergen, indicating that TLR4 signaling in epithelial cells is critical for the initiation of Th2 responses to inhaled allergens [41].

## Conclusions

Expression of TLRs on structural cells like epithelial cells and the respective functionality in terms of released mediators are important factors in the orchestration of local immune responses. We investigate the expression of all TLR receptors in primary nasal epithelial cells of healthy individuals and show an absence of TLR7 and TLR8 together with huge individual differences in mRNA expression level for TLR1-6 and TLR9. Although mRNA expression of TLRs of often taken as a measure of their activity we show that this is should be done with caution. Specific TLR agonist-induced mediator release in nasal epithelial cells is very variable between different individuals and does not correlate with the expression levels of the respective TLRs, although we show this only for a relative small number of individuals. Most notably we show that despite the presence of TLR4, CD14, and MD2 in nasal epithelial cells, stimulation with LPS does not induce any mediator response. Supporting and strengthening previous observations in nasal polyposis patients that nasal epithelial cells seem to resemble gut epithelial cells where a yet unidentified mechanism prevents epithelia routinely exposed to bacterial flora from fortuitous activation. Our data suggest that we should probably consider individual expression and activation levels better as this would affect how individuals see their microbial environment.

## Abbreviations

TLR: Toll-like receptors
PAMP: pathogen-associated molecular patterns
PRR: pattern recognition receptors
LPS: lipopolysaccharide
Poly(I:C): poly(deoxyinosinic-deoxycytidylic) acid
PGN: peptidoglycan
CpG: cytosine—phosphate—guanine
TNF-α: tumor necrosis factor-alpha
IL: interleukin
RANTES: regulated upon activation, normal T cell Expressed, and Secreted
IP-10: interferon gamma-induced protein 10
MIP-1β: macrophage inflammatory protein 1 beta
VEGF: vascular endothelial growth factor
FGF: fibroblast growth factor
G-CSF: granulocyte colony-stimulating factor
RAST: radioallergosorbent test
DC: dendritic cells
RT-PCR: realtime PCR
HBSS: Hanks’ balanced salt solution
MACS: magnetic activated cell sorting
